# The mediating role of self-regulation in the relationship between psychological resilience and computational thinking

**DOI:** 10.3389/fpsyg.2026.1714038

**Published:** 2026-04-16

**Authors:** Rumeysa Beyazhancer

**Affiliations:** Department of Mathematics Education, Bursa Uludaǧ University, Bursa, Türkiye

**Keywords:** computational thinking, mediation analysis, psychological resilience, self-regulation, teacher education

## Abstract

**Introduction:**

Psychological resilience and self-regulation are key psychological constructs that support learners' adaptation to challenges. Resilience provides the motivational foundation for persistence, while self-regulation enables planning, monitoring, and adjustment of strategies. Computational thinking (CT) represents a higher-order cognitive framework that includes creativity, algorithmic thinking, collaboration, critical thinking, and problem solving. This study examines the mediating role of self-regulation in the relationship between psychological resilience and CT among pre-service mathematics teachers.

**Method:**

Participants were 336 pre-service mathematics teachers from two state universities in Türkiye. Data were collected using the Psychological Resilience Scale, the Self-Regulation Scale, and the Computational Thinking Skills Scale. Confirmatory factor analyses supported the validity and reliability of the measures. Correlation analyses examined the associations among constructs, and mediation was tested using PROCESS Macro (Model 4) with controls for gender and grade level.

**Results:**

Psychological resilience was positively associated with CT skills (*r* = 0.34, *p* < 0.001) and self-regulation (*r* = 0.41, *p* < 0.001). Self-regulation was also positively associated with CT skills (*r* = 0.36, *p* < 0.001). Mediation analyses showed that including self-regulation reduced the associations between resilience and CT sub-dimensions, though they remained significant, indicating partial mediation. Bootstrap analyses confirmed significant indirect effects across all CT sub-dimensions.

**Discussion and conclusion:**

Findings suggest that psychological resilience is related to CT skills both directly and indirectly through self-regulation. Self-regulation acts as a mechanism that channels resilient tendencies into goal-directed strategies, thereby enhancing CT. These results underscore the value of integrating resilience, self-regulation, and CT into teacher education programs to promote adaptability and 21st-century skills.

## Introduction

In the rapidly evolving landscape of teacher education, preparing future educators requires not only cognitive skills such as problem solving, computational thinking, and metacognition but also psychological resources such as resilience and self-regulation, which together foster adaptability and sustained engagement in complex learning environments.

Self-regulation is a crucial mental skill that helps individuals manage their thoughts, feelings, and actions to achieve long-term goals (Bandura, 1991; Zimmerman, 2000). Based on social cognitive theory, self-regulation involves more than just controlling actions. It is a cycle of planning ahead, monitoring progress, and reflecting on performance (Zimmerman, 1989). In schools, self-regulation is often connected to students' ability to adapt and overcome challenges, especially in difficult learning situations (Pintrich, 2000; Boekaerts, 1999).

Psychological resilience, defined as the capacity to adapt effectively in the face of adversity, has drawn increasing attention as a protective factor that supports persistence and success in academic tasks (Masten, 2014). Resilient learners demonstrate higher motivation and perseverance, yet these outcomes often rely on their ability to engage in effective self-regulation strategies. Specifically, self-regulation acts as a bridge by enabling resilient tendencies, such as persistence and adaptability, to manifest as focused study habits and goal-directed actions. Thus, resilience alone may not directly translate into academic achievement without the mediating role of self-regulation, which channels resilient tendencies into concrete learning behaviors (de la Fuente, 2017).

At the same time, computational thinking (CT) has emerged as a fundamental 21st-century competence that involves decomposition, abstraction, pattern recognition, and algorithmic reasoning (Wing, 2006). Prior research suggests that CT is not only a set of problem-solving skills but also a metacognitive process that requires persistence, strategic planning, and self-regulated effort (Grover and Pea, 2013). In this sense, CT development may benefit from self-regulation capacities, as learners must monitor, adjust, and sustain their engagement while addressing complex computational tasks.

Within mathematics education, structured reasoning processes such as stepwise problem solving, abstraction, generalization, and symbolic representation have long been emphasized as core cognitive practices (Polya, 1957; Lithner, 2008). These processes conceptually overlap with the core components of CT, including decomposition, pattern recognition, abstraction, and algorithmic structuring (Wing, 2006; Grover and Pea, 2013; Weintrop et al., 2016). In particular, algorithmic thinking—understood as constructing systematic and rule-based solution procedures—has been associated with mathematical modeling cycles and structured problem-solving strategies (Blum and Leiss, 2007; Kaiser, 2017). From this perspective, CT in mathematics education may be considered not merely a digital competence but a cognitive framework closely related to organized forms of mathematical reasoning.

This theoretical alignment may help explain why CT skills are pedagogically meaningful for pre-service mathematics teachers. Given that mathematics teachers are expected to guide students through systematic solution pathways, facilitate transitions between mathematical representations, and structure modeling processes, algorithmic thinking and structured problem-solving skills may provide a cognitive foundation for future instructional practices. Accordingly, integrating CT into mathematics teacher education programs can be viewed as theoretically grounded within the broader framework of mathematical reasoning and modeling.

Bringing these perspectives together, this study examines the mediating role of self-regulation in the relationship between psychological resilience and CT among pre-service mathematics teachers. While psychological resilience provides the emotional foundation for persistence in challenging problem-solving contexts, CT represents a domain-specific cognitive competence that is increasingly associated with success in complex mathematical problem-solving environments. Self-regulation may serve as a critical mechanism linking these constructs, potentially translating resilience into sustained computational practices and adaptive learning behaviors.

## Psychological resilience and computational thinking

### Psychological resilience

Psychological resilience (PR) and CT represent two critical constructs that are increasingly recognized as essential for success in contemporary education and lifelong learning. The concept of resilience, derived from the Latin resilire meaning “to leap back,” refers to the individual's capacity to recover quickly from adversity or to maintain functioning in the face of stress, trauma, or significant change (Rutter, 1999; Masten, 2001; Luthar et al., 2000). In psychological research, PR is commonly conceptualized through three interrelated phenomena: (i) achieving positive outcomes despite exposure to risk, (ii) sustaining effective functioning under prolonged stress, and (iii) recovering following trauma (Masten, 2001). These dimensions highlight resilience both as a process of adaptation and as a dispositional trait that mobilizes protective factors to maintain psychological wellbeing (Bonanno et al., 2011; Feder et al., 2011; Sapienza and Masten, 2011).

### Computational thinking

CT has emerged as a foundational 21st-century competence, emphasizing the cognitive processes required to analyze complex problems, abstract patterns, and design solutions that can be implemented effectively by information-processing agents (Wing, 2006, 2011). Originally introduced into educational discourse by Papert (1980, 1993), CT has since evolved into a cross-disciplinary framework relevant not only to computer science but also to mathematics, science, and engineering education (Barr and Stephenson, 2011; Grover and Pea, 2013; Repenning et al., 2015). Its recognition in the U.S. Next Generation Science Standards (NGSS Lead States, 2013) as a central scientific practice further underlines its educational importance. Frameworks such as Brennan and Resnick's (2012) have guided the development of CT assessment tools (e.g., González et al., 2017; Mühling et al., 2015; Weintrop and Wilensky, 2015), though debates continue regarding the extent to which these tools capture computational practices and perspectives alongside computational concepts (Cutumisu et al., 2019).

Bringing these two constructs together, both PR and CT can be conceptually linked through their shared emphasis on adaptability, flexible problem solving, and persistence under uncertainty, although empirical evidence directly connecting them remains scarce (Masten, 2014; Wing, 2011). Thus, PR can be considered as a psychological foundation that fosters learners' ability to persist in and benefit from CT-oriented tasks, while CT practices, in turn, may reinforce resilience by cultivating problem-solving confidence and tolerance for ambiguity. Although these conceptual overlaps are evident, there is still a paucity of empirical research directly examining the relationship between psychological resilience and computational thinking, particularly in the context of pre-service mathematics teachers.

## Psychological resilience and self-regulation

### Self-regulation

The concept of self-regulation is fundamentally grounded in Bandura's view, which emphasizes its central role in human agency by framing it as a self-regulatory system that mediates external influences and provides the foundation for purposive action (Bandura, 1991). Building on this foundation, scholars in recent years have offered various definitions of self-regulation, and there is now consensus that it is a multidimensional construct encompassing the regulation of emotion, cognition, and behavior (Baumeister and Vohs, 2004; Cole et al., 2004; Kochanska et al., 2000; Rueda et al., 2005; Zelazo and Müller, 2002). Broadly, self-regulation refers to the deliberate modulation of one's thoughts, feelings, and actions in order to achieve more adaptive outcomes (Barkley, 2004; Carver, 2004; Grolnick and Farkas, 2002). For the purposes of this study, the concept is considered primarily in terms of intrinsic regulation, or the individual's capacity to manage their own cognitions, emotions, and behaviors (Gross and Thompson, 2007).

Psychological resilience and self-regulation are closely interconnected, as the development and use of positive self-regulation strategies play a crucial role in adapting and recovering from adversity. Self-regulation is conceptualized as a multifaceted ability shaped by temperament, developmental experiences, and attachment relationships, and it provides a critical foundation for resilience (Thomson and Jaque, 2017). In line with this view, Kauffman (2004) highlighted self-regulation as a key learner characteristic that enables individuals to plan, monitor, and adjust their strategies in order to cope effectively with challenges. Research has also emphasized that resilience cannot be understood without considering its links to emotion regulation, underscoring that both areas are conceptually related yet often overlooked (Kay, 2016). Along these lines, Troy and Mauss (2011) proposed that emotion regulation and resilience may be connected through strategies such as attentional control and cognitive reappraisal, which foster adaptive emotional responses and contribute to resilient outcomes. Recent research with adolescents further supports this link, showing that the combination of positive self-regulation and problem-focused strategies is associated with higher resilience (López-Valle et al., 2018). Consistently, studies conceptualizing self-regulation as a meta-skill have reported a positive relationship between self-regulation and resilient control, reinforcing the view that self-regulation serves as a psychological resource for resilience (de la Fuente et al., 2017a,b). However, despite this evidence, little is known about how these dynamics operate in the context of pre-service mathematics teachers, which constitutes the focus of the present study.

## Computational thinking and self-regulation

Looking through the literature, there are few studies that examine the relation between self-regulation and computational thinking. One study in early childhood education (Yang et al., 2022) found that sequencing ability and self-regulation were significant predictors of young children's CT development, with children who had lower levels of self-regulation showing greater gains in sequencing through robot programming.

More recently, Gao et al. (2023a) demonstrated that sequencing ability and self-regulation both had positive associations with CT, and that the effect of sequencing on CT was fully mediated by self-regulation. In a follow-up study, Gao et al. (2023b) further showed that CT positively predicted both sequencing ability and self-regulation after controlling for age, gender, and socioeconomic status, suggesting that CT may function as a domain-general ability grounded in broader cognitive foundations. Similarly, a study conducted in Taiwan with sixth-grade students revealed that self-regulation, along with cooperative attitude and enjoyment, significantly contributed to problem-solving skills and computational thinking, indicating that personal traits play a crucial role in shaping students' CT development (Liu et al., 2021). Yet, these relationships remain underexplored among pre-service mathematics teachers, which the present study aims to address.

## Present study

In this context, psychological factors such as resilience and self-regulation stand out as key variables that may influence pre-service math teachers' CT skills. Psychological resilience is defined as the capacity of individuals to adapt and maintain functioning in the face of stress, failure, or uncertainty (Masten, 2014). Self-regulation refers to individuals' ability to plan, monitor, and control their cognitive processes, emotions, and behaviors in order to achieve their goals (Zimmerman, 2000; Panadero, 2017). Both constructs play critical roles in learning processes and hold the potential to support the complex cognitive processes required by CT.

However, a review of the literature reveals that studies directly examining the relationship between psychological resilience and CT remain scarce. Existing research has mostly addressed these two variables indirectly. For instance, Wu et al. (2024) reported that CT education supported academic resilience through academic self-efficacy, Yusuf (2025) found that augmented reality–based project learning had parallel effects on both CT and academic resilience, and Atman Uslu (2023) examined students' academic resilience and CT profiles. Yet, these studies do not clarify the cognitive mechanisms through which resilience influences CT skills. Moreover, although conceptual overlaps such as adaptability, problem solving, and persistence under uncertainty are evident, there is still a paucity of empirical research directly testing the relationship between psychological resilience and CT (Masten, 2014; Wing, 2011).

At this point, self-regulation emerges as a critical mediating mechanism. The literature indicates that resilient individuals make more effective use of self-regulation strategies (Kauffman, 2004; de la Fuente, 2017), and that self-regulation plays a central role in the development of CT (Grover and Pea, 2013; Pasterk and Benke, 2024). Therefore, explaining the effect of resilience on CT through self-regulation fills an important gap in literature and provides a unique contribution to understanding the role of psychological resources in the development of pre-service math teachers' CT skills.

### Research problems or hypothesis

In the present study, psychological resilience was positioned as the antecedent variable based on theoretical considerations. Resilience is conceptualized as a foundational psychological resource that enables individuals to maintain motivation and emotional stability in the face of stress and uncertainty (Masten, 2014). Self-regulation, in contrast, represents the cognitive-behavioral mechanism through which these motivational resources are translated into goal-directed strategies (Bandura, 1991; de la Fuente, 2017). Accordingly, resilience is assumed to precede and facilitate self-regulatory processes, which in turn contribute to domain-specific cognitive outcomes such as CT. Although the study employs a cross-sectional design, the directional assumption of the model is grounded in established theoretical frameworks and prior empirical evidence.

**Figure 1:**
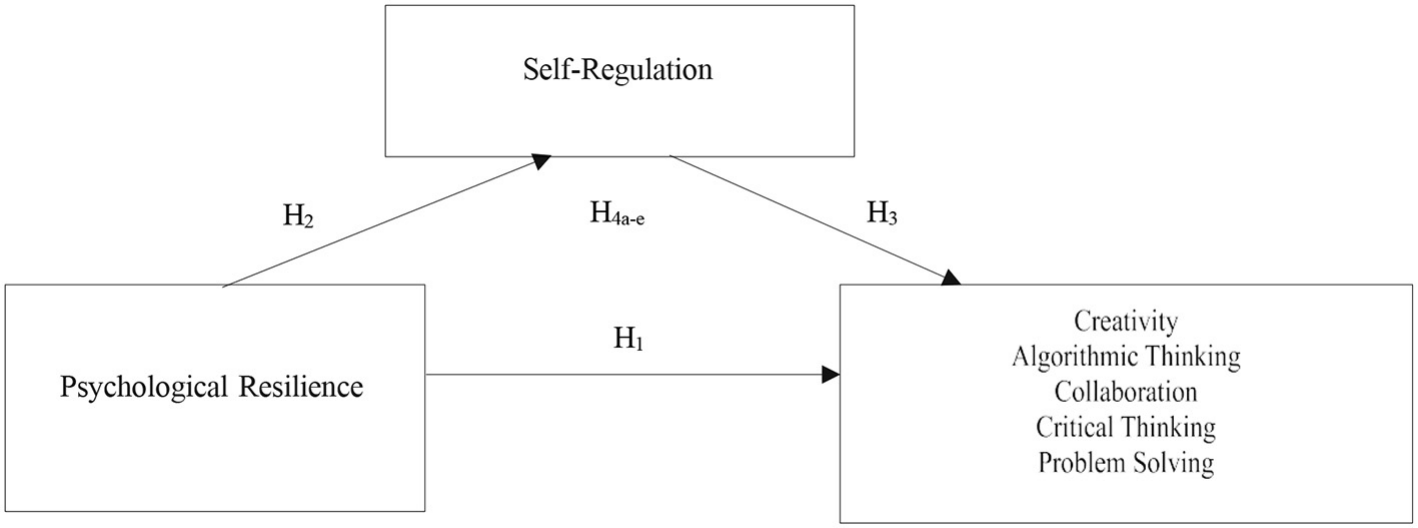
Hypothesized simple mediation model illustrating the relationship between psychological resilience and computational thinking, with the mediating role of self-regulation. Hypotheses: H1: There is a positive relationship between psychological resilience and mathematics teacher candidates' computational thinking (CT) skills, including creativity, algorithmic thinking, collaboration, critical thinking, and problem solving. H2: There is a positive relationship between psychological resilience and mathematics teacher candidates' self-regulation skills. H3: There is a positive relationship between self-regulation and mathematics teacher candidates' CT skills (creativity, algorithmic thinking, collaboration, critical thinking, and problem solving). H4 a–e: Self-regulation mediates the relationship between psychological resilience and each CT dimension—(a) creativity, (b) algorithmic thinking, (c) collaboration, (d) critical thinking, and (e) problem solving.

The aim of this study is to examine the mediating role of self-regulation in the relationship between psychological resilience and CT skills. Self-regulation represents a cognitive and behavioral mechanism that enables learners to plan, monitor, and control their learning processes, which is also closely associated with resilient behavior. While both resilience and self-regulation have independently been linked to positive learning outcomes, their combined role in shaping CT skills remains underexplored. The proposed model is presented in Figure 1.

## Materials and method

### Research design

This study employed a relational survey design to examine the mediating role of self-regulation in the relationship between psychological resilience and CT skills of mathematics teacher candidates. The relational design focuses on describing natural associations between variables without making causal claims (Fraenkel et al., 2012). The main aim was to explain how psychological resilience influences CT skills through self-regulation. To enhance the validity of the model, gender and grade level were included as control variables, ensuring more reliable identification of the relationships among independent, mediating, and dependent variables.

### Participants

The participants in the study consisted of a total of 336 teacher candidates enrolled in the mathematics teacher education program at two state universities in the Marmara region of Turkey. The participants' ages ranged from approximately 18–25. The majority of participants are female (82.7%, *n* = 278), while the rest are male (17.3%, *n* = 68). Teacher candidates are studying at different grade levels. Participants were included in the study on a voluntary basis, and informed consent was obtained prior to data collection. The reason for selecting candidates studying in the mathematics teaching program is that their computational thinking skills are closely related to mathematics education and that their psychological resilience and self-regulation skills will directly reflect in future classroom practices. In this context, it is believed that the sample in question will make a unique contribution to the study.

### Measures

#### Brief psychological resilience scale (BRS)

The Brief Psychological Resilience Scale (BRS) was developed by Smith et al. (2008) and adapted into Turkish by Dogan (2015). The scale is a unidimensional, 6-item measure developed to assess individuals' levels of recovery and adaptation in the face of difficulties. Items 2, 4, and 6 are reverse-coded. In the original study, the internal consistency coefficient was reported as α = 0.83. Since its unidimensional structure has been confirmed in previous studies, confirmatory factor analysis (CFA) was not applied in this research; only Cronbach's alpha was reported as a reliability indicator. The Cronbach's alpha coefficient calculated for the sample in this study was 0.76, indicating that the scale is reliable. Participants responded to the items using a five-point Likert scale (1 = Strongly disagree, 5 = Strongly agree).

#### Self-regulation scale for adolescents (SRSA)

The Self-Regulation Scale for Adolescents (SRSA) is a unidimensional measure developed by Kaşikci and Ögülmüş (2023) to assess individuals' goal setting, planning, emotional awareness, and problem-solving behaviors. The original form consists of 11 items. In the development study, the scale's Cronbach's alpha coefficient was reported as 0.90, McDonald's omega coefficient as 0.89, and composite reliability (CR) value as 0.89. Furthermore, CFA results supported the scale's single-factor structure (χ^2^/df = 4.55, CFI = 0.93, TLI = 0.91, SRMR = 0.041, RMSEA = 0.089). In this study, a 10-item version of the scale adapted for university level was used. During the adaptation, one item with a low factor loading was removed, strengthening the single- factor structure. The validity and reliability studies of the adapted form were repeated. The CFA results showed that the single-factor structure had acceptable fit indices (χ^2^/df = 3.20, CFI = 0.94, TLI = 0.92, SRMR = 0.048, RMSEA = 0.072). Furthermore, the Cronbach's alpha coefficient for the 10-item form was found to be 0.79, indicating that the scale is sufficiently reliable for university students.

#### Computational thinking skills scale (CTS)

The Computational Thinking Skills Scale (CTS) was developed by Korkmaz et al. (2017). The scale is used to measure pre-service teachers' computational thinking levels and consists of a total of 22 items and five dimensions (creativity, algorithmic thinking, collaboration, critical thinking, problem solving). In the development study, the scale's overall internal consistency coefficient was α = 0.86, while the coefficients for the sub-dimensions ranged from 0.65 to 0.86.

In this study, the scale was also tested using CFA based on its original five-factor structure. The analysis results showed that the five-dimensional structure of the scale had acceptable fit indices (χ^2^/df = 2.85, CFI = 0.93, GFI = 0.91, AGFI = 0.90, RMSEA = 0.062, SRMR = 0.052). The Cronbach's alpha coefficient for the scale in this sample was 0.82 overall and ranged from 0.65 to 0.78 for the subscales. Participants responded to the scale using a five-point Likert scale (1 = Strongly disagree, 5 = Strongly agree).

#### Ethical considerations

This research was conducted with the approval of the relevant university's ethics committee. Informed consent was obtained from participants prior to data collection, and detailed information was provided about the purpose and scope of the study. The confidentiality and anonymity of participants' responses were ensured, and data accessible only to the researcher were stored on a password-protected computer. The data will be retained for a specified period after completion of the study and then destroyed.

### Data collection process and data analysis

Data were collected at the institutions where teacher candidates were studying, under the supervision of the researcher, by administering the validated scales in person. The surveys took an average of 10 min to complete. Participants were included in the study on a voluntary basis after receiving preliminary information. In addition to the validity and reliability findings of the scales used in the literature, construct validity and reliability analyses were also performed on the current sample (*N* = 336). CFA was performed for the self-regulation scale using a single-factor structure, and for the CT Skills Scale using its original five-factor structure. Assumptions of normality, linearity, and homogeneity of variances were tested in preliminary analyses. Descriptive statistics were calculated for all variables, and the relationships between variables were examined using Pearson correlation analysis. Mediation analyses were conducted using bootstrapping procedures to obtain bias-corrected confidence intervals for indirect effects.

The analyses were performed using SPSS 29.0 and PROCESS macro v4.1 (Hayes, 2018). Before proceeding to hypothesis testing, the univariate normality of the variables was assessed based on skewness and kurtosis values, and all variables were found to be approximately normally distributed. Gender was dummy coded (0 = female, 1 = male) and included in all analyses as a categorical control variable. Grade level was treated as an ordinal variable and entered into the model as a continuous covariate. Both variables were included as covariates in the mediation analyses conducted using PROCESS Model 4 (Hayes, 2018). The direct and indirect effects of psychological resilience on CT skills were examined. The mediating role of self-regulation was tested using PROCESS Model 4, and bias-corrected confidence intervals were calculated based on 5,000 bootstrap samples at a 95% confidence level. The mediating effect was considered significant when zero was not included in the confidence interval.

### Findings and hypotheses tests

#### Descriptive statistics

Table 1 presents the descriptive statistics, reliability coefficients, and correlations among the variables analyzed in the study. To assess the assumption of normality, skewness and kurtosis values were examined. According to George and Mallery (2010), values within the range of ±2 indicate an approximately normal distribution. As shown in Table 1, the skewness and kurtosis values for all variables fall within this acceptable range, confirming the assumption of normality.

**Table 1:** Descriptive and correlations results of study variables.

Variables	*X*	SD	Skewn.	Kurtosis	Cronbach	1	2	3	4	5	6
1. Psychological resilience (PR)	3.32	0.61	0.111	−0.445	0.755	—					
2. Self-regulation (SR)	3.82	0.47	348	−0.751	0.788	0.469^**^	—				
3. Creativity (CR)	3.92	0.52	−0.386	0.632	0.779	0.339^**^	0.477^**^	—			
4. Algorithmic thinking (AT)	3.89	0.45	0.016	−0.360	0.715	0.444^**^	0.544^**^	0.761^**^	—		
5. Collaboration (CO)	3.94	0.46	−0.034	0.125	0.743	0.366^**^	0.548^**^	0.677^**^	0.704^**^	—	
6. Critical thinking (CT)	3.83	0.43	−0.354	0.943	0.652	0.521^**^	0.560^**^	0.580^**^	0.579^**^	0.518^**^	—
7. Problem solving (PS)	3.81	0.47	−0.240	0.720	0.656	0.438^**^	0.494^**^	0.508^**^	0.736^**^	0.577^**^	0.467^*^

^*^indicates statistical significance (*p* < 0.05). ^**^Correlation is significant at the 0.01 level (two-tailed).

The mean score for psychological resilience was 3.32 (SD = 0.61), while self-regulation had a mean of 3.82 (SD = 0.47). Regarding the sub-dimensions of computational thinking, the mean scores were as follows: creativity 3.92 (SD = 0.52), algorithmic thinking 3.89 (SD = 0.45), cooperation 3.94 (SD = 0.46), critical thinking 3.83 (SD = 0.43), and problem solving 3.81 (SD = 0.47). Furthermore, the reliability analysis showed that Cronbach's alpha values ranged between 0.65 and 0.79, indicating acceptable internal consistency for the scales in this study. Correlations among all variables are presented in Table 1.

The results of the correlation analysis indicate that there are significant relationships between the variables included in the study. Within the scope of H1, positive and significant relationships were found between psychological resilience and all sub- dimensions of information processing thinking. Psychological resilience was positively and significantly correlated with creativity (*r* = 0.339, *p* ≤ 0.01), algorithmic thinking (*r* = 0.444, *p* ≤ 0.01), collaboration (*r* = 0.366, *p* ≤ 0.01), critical thinking (*r* = 0.521, *p* ≤ 0.01), and problem solving (*r* = 0.438, *p* ≤ 0.01). These findings reveal that students with high psychological resilience demonstrate higher levels of skill in all dimensions of cognitive processing. Under H2, a significant relationship was also found between psychological resilience and self-regulation (*r* = 0.469, *p* ≤ 0.01). This result indicates that students with high psychological resilience also have stronger self-regulation skills. Under H3, positive and significant relationships were found between self-regulation and the sub- dimensions of computational thinking. Self-regulation was positively correlated with creativity (*r* = 0.477, *p* ≤ 0.01), algorithmic thinking (*r* = 0.544, *p* ≤ 0.01), collaboration (*r* = 0.548, *p* ≤ 0.01), critical thinking (*r* = 0.560, *p* ≤ 0.01), and problem solving (*r* = 0.494, *p* ≤ 0.01). These findings indicate that students with high self-regulation skills are more successful in all dimensions of information processing thinking.

According to the correlation analysis results, as psychological resilience increases, both self-regulation and CT skills increase; as the level of self-regulation rises, all sub-dimensions of CT also strengthen. In line with these findings, hypotheses H1, H2, and H3 were supported. After confirming these meaningful relationships between variables, mediation analysis was conducted to test the mediating role of self-regulation in the effect of psychological resilience on cognitive processing. Findings were obtained form the basis for hypothesis H4 in this regard.

#### Mediation analysis results

The mediating effect of psychological resilience on CT skills (creativity, algorithmic thinking, collaboration, critical thinking, and problem solving) through self-regulation was analyzed by controlling gender and class level variables.

**Figure 2:**
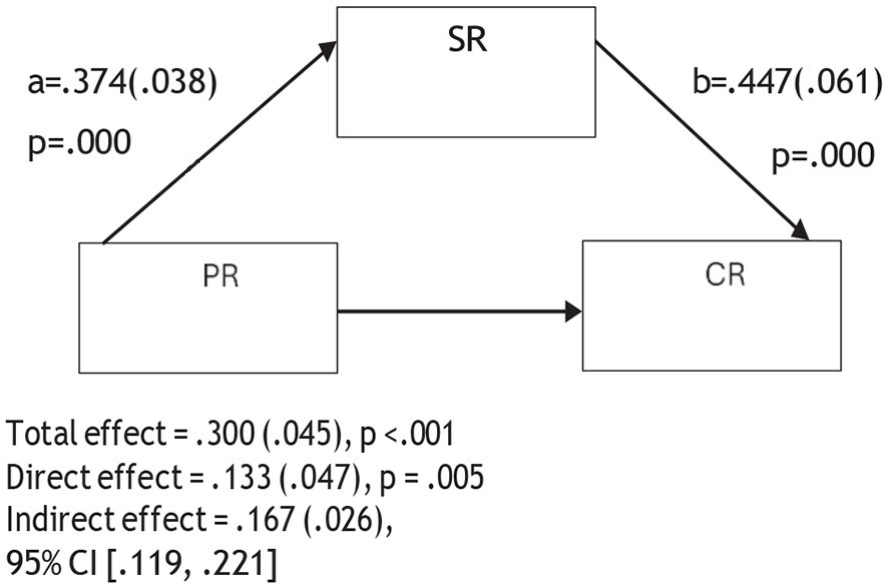
Mediation model of the relationship between psychological resilience and creativity through self-regulation.

**Figure 3:**
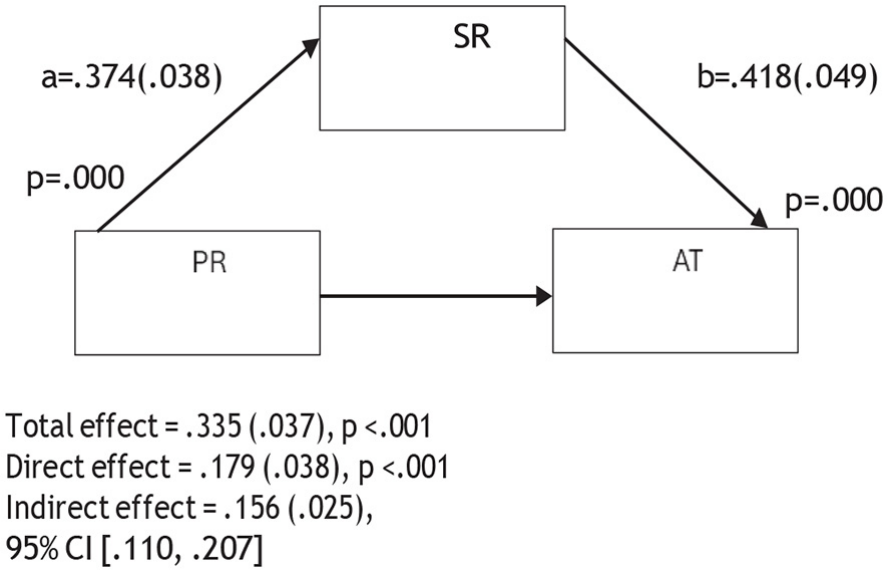
Mediation model of the relationship between psychological resilience and algorithmic thinking through self-regulation.

**Figure 4:**
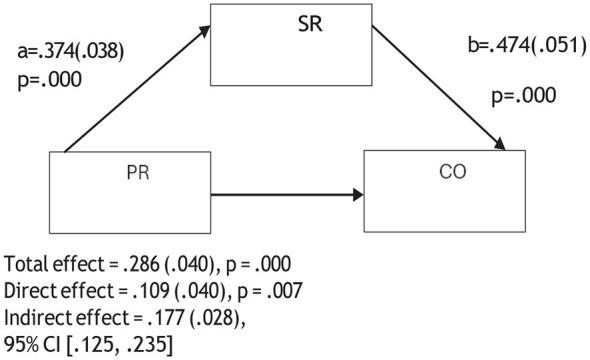
Mediation model of the relationship between psychological resilience and cooperation through self-regulation.

**Figure 5:**
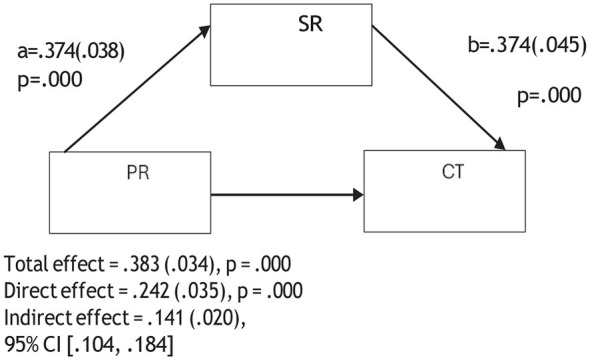
Mediation model of the relationship between psychological resilience and critical thinking through self-regulation.

**Figure 6:**
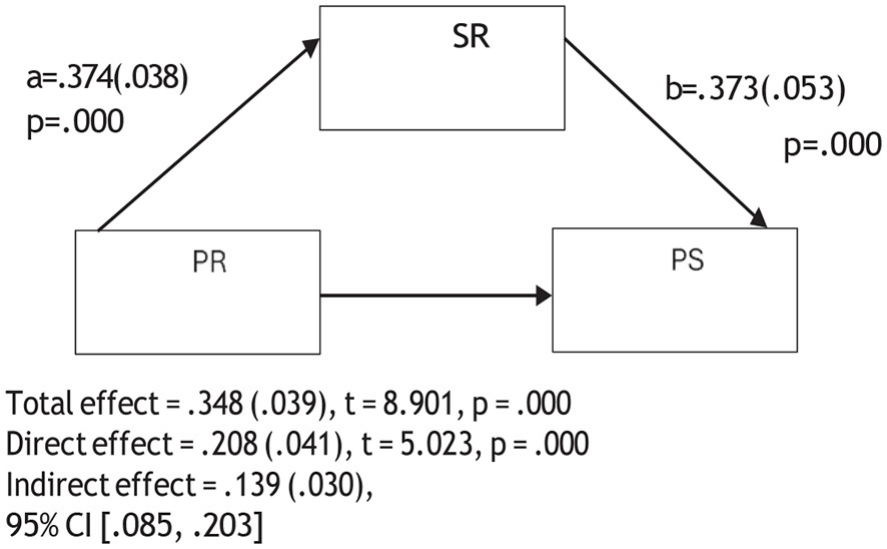
Mediation model of the relationship between psychological resilience and problem solving through self-regulation.

An examination of Table 2 and Figures 2–6 reveals that psychological resilience has a direct, positive, and statistically significant effect on all sub-dimensions of CT. In addition, self-regulation is significantly associated with psychological resilience (β = 0.374, *p* < 0.001) and shows positive and significant effects on creativity (β = 0.447, *p* < 0.001), algorithmic thinking (β = 0.418, *p* < 0.001), cooperation (β = 0.474, *p* < 0.001), critical thinking (β = 0.377, *p* < 0.001), and problem solving (β = 0.373, *p* < 0.001).

**Table 2:** Analysis of mediating effects of self-regulation.

Overall fit index										
Variable	Predictor	95% CI								
		*R*	*R* ^2^	*F*	β	SE	*t*	*p*-value	LLCI	ULCI
Self-regulation	Sex	0.480	0.230	32.844	−0.090	0.058	−1.567	0.118	−0.204	0.023
	Grade				0.052	0.040	1.298	0.195	−0.027	0.130
	Psychological resilience				0.374	0.038	9.838	0.000	0.299	0.448
Creativity	Sex	0.496	0.246	26.813	−0.037	0.064	−0.588	0.557	−0.163	0.088
	Grade				0.025	0.044	0.583	0.561	−0.061	0.112
	Psychological resilience				0.133	0.047	2.806	0.005	0.040	0.227
	Self-regulation				0.447	0.061	7.366	0.000	0.327	0.566
Algorithmic thinking	Sex	0.586	0.343	42.867	0.008	0.052	0.159	0.874	−0.093	0.110
	Grade				−0.032	0.035	−0.901	0.368	−0.102	0.038
	Psychological resilience				0.179	0.038	4.655	0.000	0.103	0.255
	Self-regulation				0.418	0.049	8.515	0.000	0.322	0.515
Cooperation	Sex	0.566	0.320	38.594	0.021	0.054	0.388	0.698	−0.085	0.127
	Grade				0.056	0.037	1.495	0.136	−0.018	0.129
	Psychological resilience				0.109	0.040	2.701	0.007	0.030	0.188
	Self-regulation				0.474	0.051	9.205	0.000	0.372	0.575
Critical thinking	Sex	0.638	0.408	56.407	−0.054	0.047	−1.142	0.254	−0.146	0.039
	Grade				−0.061	0.032	−1.888	0.060	−0.125	0.003
	Psychological resilience				0.242	0.035	6.872	0.000	0.173	0.311
	Self-regulation				0.377	0.045	8.401	0.000	0.289	0.466
Problem solving	Sex	0.548	0.300	35.210	−0.026	0.056	−0.463	0.644	−0.135	0.084
	Grade				−0.035	0.038	−0.905	0.366	−0.110	0.041
	Psychological resilience				0.208	0.041	5.023	0.000	0.127	0.290
	Self-regulation				0.373	0.053	7.046	0.000	0.269	0.477

The mediation analyses showed that when self-regulation was included in the model, the association between psychological resilience and the sub-dimensions of CT were reduced but remained statistically significant. For instance, the association between psychological resilience and creativity decreased from 0.300 to 0.133, algorithmic thinking from 0.335 to 0.179, cooperation from 0.286 to 0.109, critical thinking from 0.383 to 0.242, and problem solving from 0.348 to 0.208. These reductions suggest that self-regulation is partially involved in the relationship between psychological resilience and the sub- dimensions of CT. Therefore, Hypothesis 4 was supported. In other words, psychological resilience was related to pre-service teachers' CT skills both directly and indirectly through self-regulation.

Figures 2–6 are mediation models demonstrating the mediating role of self-regulation in the effects of psychological resilience on the sub-dimensions of computational thinking (creativity, algorithmic thinking, cooperation, critical thinking, and problem solving).

## Discussion

### Psychological resilience and computational thinking

The analyses revealed that psychological resilience was positively and significantly associated with all sub-dimensions of CT, including creativity, algorithmic thinking, collaboration, critical thinking, and problem solving. These findings suggest that psychological resilience, as a psychological resource, may support students' cognitive processes by enabling them to sustain persistence and flexibility when engaging with cognitively demanding tasks such as computational problem solving. In particular, resilient pre-service mathematics teachers may be better equipped to cope with the uncertainty and complexity inherent in CT practices, allowing them to sustain engagement and generate adaptive strategies.

These findings align with earlier research linking resilience to broader academic outcomes. For instance, Wu et al. (2024) showed that CT education boosted students' academic resilience through academic self-efficacy, while Yusuf (2025) found that augmented reality–based project learning had similar effects on both CT and academic resilience. Likewise, Atman Uslu (2023) examined students' academic resilience and CT, revealing that these two constructs are often interconnected in educational settings. However, these studies mainly focused on resilience at the academic level and did not clarify how resilience directly relates to the specific cognitive processes involved in CT.

By focusing explicitly on the relationship between psychological resilience and CT in pre-service mathematics teachers, the present study extends this line of inquiry. Our findings provide direct empirical evidence that resilience contributes to CT competence, moving beyond general academic outcomes to demonstrate its role in shaping specific sub-skills such as creativity and algorithmic reasoning. This advances the literature by suggesting that resilience may function as an underlying psychological foundation that fosters learners' ability to persist in and benefit from CT-oriented tasks. At the same time, given the scarcity of studies addressing this relationship directly, our findings highlight the need for further research to disentangle the pathways through which resilience supports CT development in teacher education contexts.

### Psychological resilience and self-regulation

The findings of this study indicated that psychological resilience had a significant positive effect on self-regulation, suggesting that resilient pre-service mathematics teachers are more capable of setting goals, planning, and regulating their emotions and behavior effectively. This result also implies that resilient individuals may be more successful in managing self-regulatory processes such as goal setting, planning, and emotional regulation. This aligns with Bandura's (1991) social cognitive theory, which framed self-regulation as a central system of human agency that mediates external influences and underpins purposive action. In line with this perspective, our results confirm that resilience can provide the motivational and emotional resources necessary for activating self-regulatory processes.

These findings are also consistent with prior research emphasizing the close interconnection between resilience and self-regulation. For instance, Thomson and Jaque (2017) conceptualized self-regulation as a foundational capacity for resilience, while Kauffman (2004) identified it as a key learner characteristic enabling individuals to plan, monitor, and adjust their strategies to cope with challenges. Similarly, López-Valle et al. (2018) showed that adolescents who combined positive self-regulation strategies with problem-focused approaches demonstrated higher levels of resilience, reinforcing the idea that self-regulation is not only a correlation but also a mechanism that supports adaptive adjustment. Complementary evidence from de la Fuente et al. (2017a,b) further highlighted self-regulation as a meta-skill, positively associated with resilient control, thereby strengthening the argument that self-regulation functions as a psychological resource that channels resilience into concrete learning behaviors.

By demonstrating this link in the context of pre-service mathematics teachers, the present study expands the scope of previous research, which has primarily focused on adolescents or general student populations. The findings suggest that resilience may enhance teacher candidates' ability to engage in effective self-regulated learning, a capacity that is vital not only for their own academic success but also for their future roles in supporting students' learning processes.

### Self-regulation and computational thinking

The research findings revealed that self-regulation was significantly associated with all sub-dimensions of CT: creativity, algorithmic thinking, collaboration, critical thinking, and problem solving. These results indicate that self-regulation skills play a central role in enabling students to exercise cognitive control, develop strategies, and manage problem-solving processes more effectively. Literature similarly emphasizes that self-regulation is a critical determinant for the development of higher-order cognitive skills. Therefore, this finding supports the strong theoretical link between self-regulation and computational thinking.

This result underscores the idea that learners who effectively plan, monitor, and adjust their learning processes are better positioned to engage in the complex cognitive tasks required by CT. These results are consistent with previous research highlighting the role of self-regulation in CT development. For example, Yang et al. (2022) found that sequencing ability and self-regulation were significant predictors of CT development in young children, suggesting that early cognitive control processes are crucial for computational learning. Gao et al. (2023a) extended this work by showing that sequencing ability influences CT indirectly through self-regulation, further supporting the mediating role of self-regulatory processes. In a subsequent study, Gao et al. (2023b) reported that CT itself positively predicted both sequencing and self-regulation, indicating reciprocal relationships and reinforcing the view that CT may function as a domain-general competence rooted in broader cognitive foundations. Similarly, Liu et al. (2021) demonstrated with Taiwanese sixth-grade students that self-regulation, along with cooperative attitude, learning style, and enjoyment, significantly contributed to problem-solving and CT, highlighting the importance of personal traits for CT development.

Taking together, these studies provide converging evidence that self-regulation is not only an important predictor but also a potential mediator in the development of CT. The present study extends this body of knowledge by demonstrating similar associations in the context of pre-service mathematics teachers, a group for whom CT skills are essential for both professional development and the effective integration of CT into mathematics education. By confirming the robust link between self-regulation and CT in this context, our findings emphasize the need to strengthen self-regulatory capacities in teacher preparation programs as a pathway to fostering CT competence.

### The mediating role of self-regulation in the relationship between psychological resilience and computational thinking outcomes

Mediation analyses revealed that self-regulation partially mediated the relationship between psychological resilience and different sub-dimensions of CT. When self-regulation was included in the model, the direct effects of resilience on CT decreased, while the indirect effects remained significant across all sub-dimensions. This finding clarifies the mechanism through which resilience translates into enhanced CT competencies: resilient tendencies provide the motivational and emotional foundation, while self-regulation channels these tendencies into goal-directed strategies that facilitate CT development.

This result fills an important gap in existing literature. Wu et al. (2024) and Yusuf (2025) identified links between CT and resilience via other mediators such as self-efficacy, but they did not explicitly test self-regulation as a mechanism. The present study contributes by confirming self-regulation as a critical pathway, supporting earlier claims that self-regulation serves as a bridge between cognitive and affective constructs (de la Fuente et al., 2017a,b). By providing empirical evidence of this mediating role, the study advances our understanding of how psychological resilience influences CT, particularly in the context of pre-service mathematics teachers, and highlights the importance of fostering self-regulatory capacities in teacher education programs.

## Conclusion

This study examined the mediating role of self-regulation in the relationship between psychological resilience and CT among pre-service mathematics teachers. The findings confirmed that both resilience and self-regulation were positively associated with CT sub-dimensions such as creativity, algorithmic thinking, collaboration, critical thinking, and problem solving. Furthermore, resilience was found to predict self-regulation, and mediation analyses demonstrated that self-regulation partially explained the link between resilience and CT. These results suggest that resilience provides the motivational and emotional foundation for persistence, while self-regulation transforms these tendencies into goal-directed behaviors that directly enhance CT development. In practice, this means that when pre-service mathematics teachers face setbacks—such as struggling to debug a programming task or feeling uncertain about how to decompose a complex mathematical problem—resilience enables them to stay motivated and avoid disengagement. However, it is through self-regulation that this motivation is channeled into concrete strategies: planning alternative approaches, monitoring their problem-solving steps, seeking feedback from peers, and adjusting methods when initial attempts fail. In classroom contexts, these combined processes not only foster persistence in computationally demanding tasks but also build confidence and problem-solving efficiency, highlighting self-regulation as the mechanism that operationalizes resilience into measurable CT outcomes.

### Theoretical and practical contributions

The study makes several contributions to literature. First, it provides direct empirical evidence of the connection between psychological resilience and CT, which has been discussed conceptually but rarely tested. Second, by identifying self-regulation as a mediating mechanism, the study advances theoretical understanding of how affective and cognitive factors interact to support CT. Third, the focus on pre-service mathematics teachers extends the scope of prior research, highlighting the importance of psychological resources in teacher preparation. Practically, the findings suggest that fostering resilience and self-regulation in teacher education programs may enhance future teachers' CT competence and their ability to integrate CT into mathematics instruction.

### Limitations and future directions

Despite its contributions, the study has several limitations. The use of self-report measures may have introduced bias, and future research could incorporate performance-based or observational assessments. The sample was limited to pre-service mathematics teachers from a specific context, which may restrict the generalizability of the findings. In addition, cross-sectional design limits the ability to draw causal inferences about the relationships between resilience, self-regulation, and CT.

In addition, the gender distribution of the sample was predominantly female, which may limit the generalizability of the findings across more gender-balanced populations. Future research could examine whether the relationships among psychological resilience, self-regulation, and CT differ across gender groups.

Future research should build on these findings in several ways. Longitudinal or experimental designs could provide stronger evidence for the causal mechanisms linking resilience, self-regulation, and CT. Expanding the sample to include pre-service teachers from other disciplines or practicing teachers could further validate the findings across educational contexts. Finally, interventions designed to strengthen resilience, and self-regulation could be tested for their impact on CT, providing practical implications for curriculum design in teacher education.

## Data Availability

The raw data supporting the conclusions of this article will be made available by the authors, without undue reservation.
